# The yeast Aft2 transcription factor determines selenite toxicity by controlling the low affinity phosphate transport system

**DOI:** 10.1038/srep32836

**Published:** 2016-09-13

**Authors:** María Pérez-Sampietro, Albert Serra-Cardona, David Canadell, Celia Casas, Joaquín Ariño, Enrique Herrero

**Affiliations:** 1 Departament de Ciències Mèdiques Bàsiques, Universitat de Lleida, IRBLleida, 25198-Lleida, Spain; 2 Institut de Biotecnologia i Biomedicina and Departament de Bioquímica i Biologia Molecular, Universitat Autònoma de Barcelona, 08193-Cerdanyola del Vallès, Barcelona, Spain

## Abstract

The yeast *Saccharomyces cerevisiae* is employed as a model to study the cellular mechanisms of toxicity and defense against selenite, the most frequent environmental selenium form. We show that yeast cells lacking Aft2, a transcription factor that together with Aft1 regulates iron homeostasis, are highly sensitive to selenite but, in contrast to *aft1* mutants, this is not rescued by iron supplementation. The absence of Aft2 strongly potentiates the transcriptional responses to selenite, particularly for DNA damage- and oxidative stress-responsive genes, and results in intracellular hyperaccumulation of selenium. Overexpression of *PHO4*, the transcriptional activator of the *PHO* regulon under low phosphate conditions, partially reverses sensitivity and hyperaccumulation of selenite in a way that requires the presence of Spl2, a Pho4-controlled protein responsible for post-transcriptional downregulation of the low-affinity phosphate transporters Pho87 and Pho90. *SPL2* expression is strongly downregulated in *aft2* cells, especially upon selenite treatment. Selenite hypersensitivity of *aft2* cells is fully rescued by deletion of *PHO90*, suggesting a major role for Pho90 in selenite uptake. We propose that the absence of Aft2 leads to enhanced Pho90 function, involving both Spl2-dependent and independent events and resulting in selenite hyperaccumulation and toxicity.

Selenium (Se) is an essential element for life as component of selenocysteine in selenoproteins^1^. Some of these are enzymes protecting against oxidation of macromolecules by reactive oxygen species. However, high concentrations of Se become toxic to the cells, particularly in the form of selenide, which forms from environmental selenite^2^,^3^. Since *Saccharomyces cerevisiae* lacks selenoproteins, this yeast species is employed to study the toxicity effects of Se (and its molecular derivatives) without interference from its requirement as growth factor as occurs in animal cells^2^,^3^. Studies in this microorganism showed that exposure to high concentrations of selenite generates oxidative stress conditions and causes DNA damage, depletion of reduced glutathione and irreversible protein oxidation^4^,^5^,^6^,^7^,^8^.

In glucose-containing media selenite enters yeast cells through the phosphate symporters^9^, while with other carbon sources the monocarboxylic acids transporter Jen1 contributes to selenite import^10^. Two phosphate transport systems operate in *S. cerevisiae*^11^. The high affinity system is composed of Pho84 and Pho89, and functions at both low and high phosphate concentrations. *PHO84* and *PHO89* gene expression is upregulated at low phosphate concentration by the Pho4 transcription factor, depending on the *PHO* pathway^12^. The Pho84 transporter operates preferentially at neutral and acidic pH, while Pho89 is functional at alkaline pH. On the other hand, the low affinity transport system operates at higher phosphate concentrations (>1 mM), is composed by Pho87 and Pho90, and in response to low phosphate conditions it is post-transcriptionally downregulated by means of vacuolar targeting by Spl2 (also a member of the *PHO* regulon)^13^,^14^. At moderate and high phosphate concentration, selenite enters through Pho87 and Pho90, which discriminate poorly between selenite and phosphate, while at low phosphate Pho84 is the main selenite symporter^9^.

Studies on the transcriptional response of selenite-treated *S. cerevisiae* cells showed upregulation of functional gene categories such as those involved in iron homeostasis and in general stress and protein degradation responses^15^. In yeast, iron homeostasis is under the control of the Aft1 and Aft2 transcription factors, that regulate the expression of genes belonging to the so called iron regulon, Aft1 playing the primary role in such function^16^,^17^. In spite of the overlapping roles of Aft1 and Aft2 in the response to changes in iron availability^18^,^19^, both factors may have some independent functions, in accordance with the slight differences between the DNA motifs they recognize^20^. Thus, although in iron-minus conditions most of the genes of the iron regulon are primarily controlled by Aft1, in these conditions Aft2 is the direct regulator of expression of *MRS4* and *SMF3*, respectively involved in iron transport into mitochondria and vacuoles^20^. In addition, studies with the full collection of *S. cerevisiae* null mutants revealed that Aft1 and Aft2 could additionally participate in other functions different from iron homeostasis regulation^21^. Concerning Aft2, this is the primary regulator of *GEX1* expression, whose protein product accumulates under iron depletion although it carries out iron-independent functions as a glutathione exchanger at the vacuolar and plasma membranes^22^.

Based on mutant phenotype studies, the physiological function of Aft1 has been demonstrated also in the defence against diverse environmental stresses, although only in some cases the hypersensitivity of the Aft1-defective mutant could be related to interference of the stress conditions on iron uptake or metabolism^23^,^24^,^25^,^26^,^27^,^28^. The contributing role of Aft2 in parallel to Aft1 to stress defence has been demonstrated in the case of hydrogen peroxide^18^,^29^ and hydroxyurea, an inhibitor of ribonucleotide reductase causing DNA replication stress^30^, as deduced from the additive effect of the mutations and the rescue of the sensitive phenotype of the double mutant by iron supplementation.

The upregulation of genes for intracellular iron mobilization and high-affinity iron uptake upon selenite treatment of *S. cerevisiae* cells suggests a role for Aft1 (and perhaps Aft2) in selenite toxicity. In fact, cells lacking Aft1 are hypersensitive to selenite and this phenotype is rescued by iron addition to the medium^31^, indicating interference between selenite effects and iron homeostasis in yeast cells. Here, we study the relationship between Aft2 and selenite toxicity, and show that the hypersensitivity of cells lacking Aft2 to this agent cannot be rescued by iron supplementation and that it is due to increased accumulation of selenite inside cells through the low affinity phosphate transport system. Thus, the present study demonstrates a relationship between the Aft2 factor and phosphate and selenite transporters, and contributes to explain the mechanisms through which Se exerts its toxic effects.

## Results

### The absence of Aft2 provokes selenite hypersensitivity in an iron-independent manner

**Figure 1:**
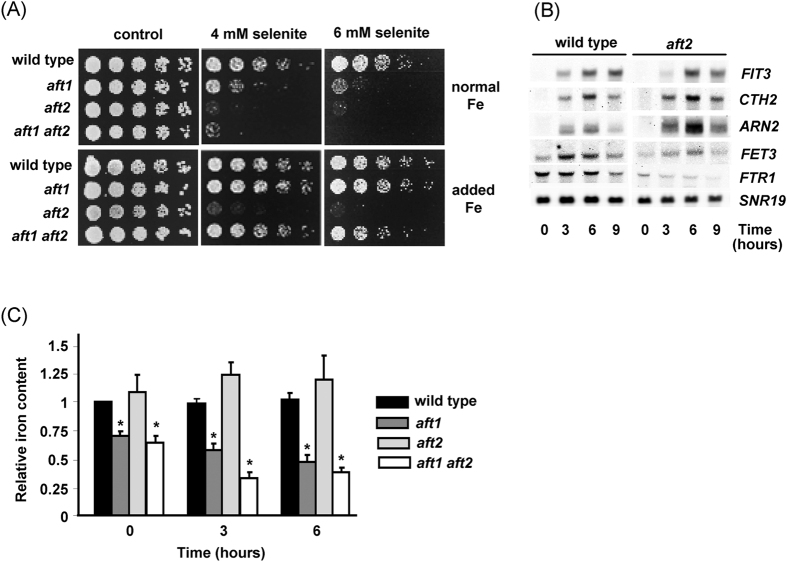
The *aft2* mutant is hypersensitive to selenite. (**A**) Exponential cultures of the following strains in YPD medium were serially diluted and spotted on YPD plates containing sodium selenite: wild type (W303-1A), *aft1* (MML348), *aft2* (MML1086) and *aft1aft2* (MML1088). Plates contained no additional iron (normal iron) or were added with 90 μM bathophenanthroline sulphonic acid plus 100 μM ferrous sulphate (added iron). Growth was recorded after 2 days of incubation at 30 °C. (**B**) Time-course analysis by Northern blot of expression of the indicated genes in wild type and *aft2* cells grown in YPD medium after selenite addition (2 mM, final concentration) at time 0. *SNR19* is employed as loading control. (**C**) Relative intracellular iron content in the same strains employed in panel (**A**), at the indicated times after selenite addition (6 mM, final concentration in YPD medium). Values are made relative to those of wild type cells at time 0. Bars represent the mean of three independent experiments (±SD). (Turkey-Kramer test, *p < 0.05 when compared with the wild type value at the respective treatment time).

To comparatively evaluate the relevance of Aft1 and Aft2 in selenite tolerance we grew wild type, and *aft1 and aft2* mutants in the presence of different concentrations of selenite. Cells lacking Aft2 were even more sensitive to this agent than those lacking Aft1 (Fig. 1A). As already described^31^, addition of iron to the medium eliminated the sensitivity to selenite of *aft1* cells. On the contrary, the *aft2* mutant was still hypersensitive to the agent in the presence of iron supplement. Remarkably, addition of iron significantly improved growth of the *aft1aft2* strain the presence of selenite (Fig. 1A). These results support that Aft1 and Aft2 differ in their protective role against selenite and that, in the case of Aft2, this role seems to be unrelated to the iron homeostatic mechanisms. We then analyzed whether the observed activation of genes of the iron regulon upon selenite treatment^15^ was dependent on Aft2. Northern blot experiments were done at a lower selenite concentration (2 mM) than the growth phenotype experiments to avoid potential direct effects of selenite on RNA integrity. These are similar conditions to those employed in previous studies^8^,^31^. Statistical analyses (Turkey-Kramer test) of the Northern blot data revealed no significant differences in expression of *FIT3*, *CTH2* or *ARN2* (Fig. 1B) between wild type and *aft2* mutant cells at equivalent treatment times. On the contrary, expression of other genes of the regulon that are modestly or not activated at all, such as *FET3* or *FTR1*, could be up to some extent dependent on Aft2 (Fig. 1B).

Selenite did not cause significant changes in intracellular iron levels in wild type or in *aft2* cells (Fig. 1C). In contrast, the *aft1* mutant and the double mutant already displayed lower intracellular iron levels than wild type cells before selenite treatment, and these levels further decreased upon treatment. These observations fit well with the protective effect of iron addition to the medium to the *aft1* and *aft1aft2* strains (Fig. 1A), and add support to the iron-independent role of Aft2 in protection against selenite toxicity. Since *GEX1* is an Aft2 target not related to iron homeostasis, we determined selenite sensitivity in yeast cells lacking *GEX1* and/or its paralogue *GEX2*. Neither the single mutants nor the double mutant were sensitive to selenite (Supplementary Fig. S1), discarding the *GEX1/2* genes as possible effectors of Aft2 in relation to protection against selenite.

### Genes involved in the oxidative stress response and in DNA metabolism are overexpressed in selenite-treated *aft2* cells

To analyze how Aft2 could influence selenite sensitivity in yeast, we carried out a genome-wide transcriptomic study in wild type and *aft2* cells treated with this agent. A sublethal dose of selenite (1 mM), smaller than that used in the experiments described above, was employed to avoid interference with growth inhibitory effects, and the transcriptomic profiles were determined at early (1 h), medium (3 h) and advanced (5 h) treatment times.

Comparison of the profiles of wild type and *aft2* cells in the absence of selenite stress showed relatively minor changes. Only 18 genes were induced over 2-fold as a result of the absence of the transcription factor, with no obvious common GO signature, whereas the mRNA level of 45 genes decreased by at least 0.5-fold (including *AFT2*). In this case this set was enriched in genes related to transmembrane transport (*p* < 2.7E-6), including *OPT2*, *GAP1*, *PHO89*, *SUL1* and *SUL2*. Other targets of the *PHO* regulon, such as *PHO5* and *PHO12* were also somewhat repressed.

**Figure 2:**
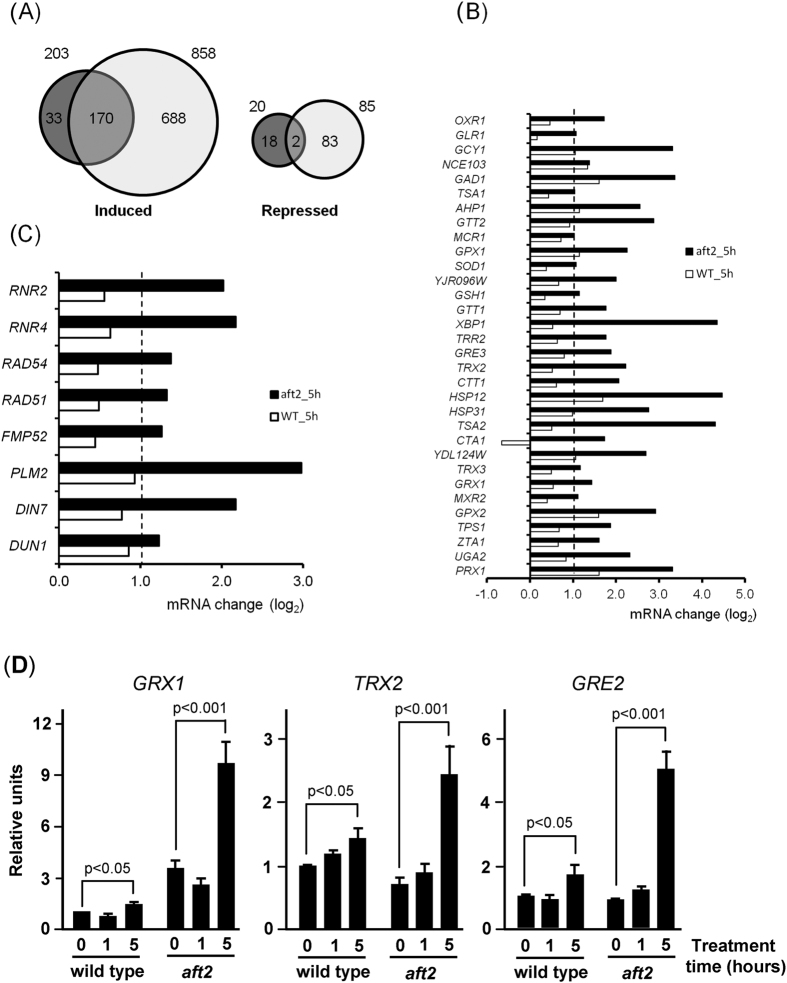
Transcriptomic profiling by RNA-Seq of wild type and *aft2* cells treated with 1 mM sodium selenite. (**A**) A total number of 5415 genes with valid data for both the wild-type (dark grey circle) and the *aft2* (light grey circle) strains subjected to 1 mM selenite treatment were evaluated. Genes showing induction or repression in both strains are shown as intersection of the circles. The number of genes in each category is indicated. Circles are not drawn to scale. (**B**) Changes in mRNA levels (log_2_ scale) of selected genes known to respond to oxidative stress in the wild type (open bars) and *aft2* (closed bars) strains after 5 h of treatment with selenite. (**C**) Changes in mRNA levels for genes known to specifically respond to DNA damage^60^. See main text for details. (**D**) Exponential cultures in SC medium of wild type and *aft2* cells transformed with reporter plasmids in which the respective gene promoter was fused to *lacZ* were treated from time 0 with 1 mM selenite. β-galactosidase activity was measured in samples at the indicated times, and for each gene the values were made relative to the respective value in untreated wild type cells. Data are expressed as mean ± SD from three independent experiments.

The effect of selenite was studied for a total number of 5415 genes with valid data for both wild type and *aft2* strains. Data clustering analysis (Supplementary Fig. S2) clearly illustrates that mutation of *AFT2* strongly potentiates the transcriptional changes induced by selenite. In the wild type strain, a total number of 203 genes showed an increase in mRNA levels in at least one time point after selenite addition (Fig. 2A), including genes involved in oxidation-reduction processes (*p* < 1.7E-8), glycogen metabolism (*p* < 2.1E-5) and response to oxidative stress (*p* < 8.4E-5). Only 20 genes were found to be repressed by selenite, with no specific GO enrichment pattern. In contrast, 858 genes became induced in the *aft2* strain, among which genes involved in oxidation-reduction processes (*p* < 1.15E-30) and in the response to oxidative stress (*p* < 8.9E-14) were abundantly represented. One hundred seventy genes were induced at least 2-fold by selenite in both the wild type and *aft2* strains (Fig. 2A). However, with very few exceptions, induction was much more intense in the *aft2* strain, as deduced from the comparison of the median of the changes for this subset of genes (2.43-fold for the wild type *vs* 4.71 for the *aft2* strain). We found 85 genes whose expression was repressed in the *aft2* strain exposed to selenite. Among the repressed genes there was an excess of those involved in cell separation after cytokinesis (*p* < 2.6E-6) and related to the fungal cell wall (*p* < 8.7E-9).

As observed in our clustering analysis (Supplementary Fig. S2), cluster number 8 is enriched in genes involved in the cellular response to oxidative stress (*p* < 1.4E-6). We crossed our data with a set of genes known to respond to oxidative stress that was generated by combining genes under Gene Ontology annotation GO:0034599 and those reported by Morano *et al*.^32^. Figure 2B shows the expression profile after 5 h of treatment with selenite of 32 selected genes with a protective role against oxidative stress. Only in a few cases these genes are induced in the wild type strain and, even in these cases, the change in the mRNA level is rather modest (average value of 1.80-fold). In contrast, these genes are potently induced by selenite in the *aft2* strain (average value of 6.05-fold). We subsequently validated these results for some selected genes (*GRX1*, *TRX2* and *GRE2*) in time-course studies by employing constructions in which the *lacZ* reporter was fused to the promoter of the respective tested gene (Fig. 2D). These results confirm that transcriptional upregulation is potently enhanced in the mutant compared to the wild type upon selenite treatment, and supports that the oxidative stress conditions provoked by selenite are more intense in the absence of Aft2.

### Aft2-deficient cells display an increased DNA damage response upon selenite treatment

**Figure 3:**
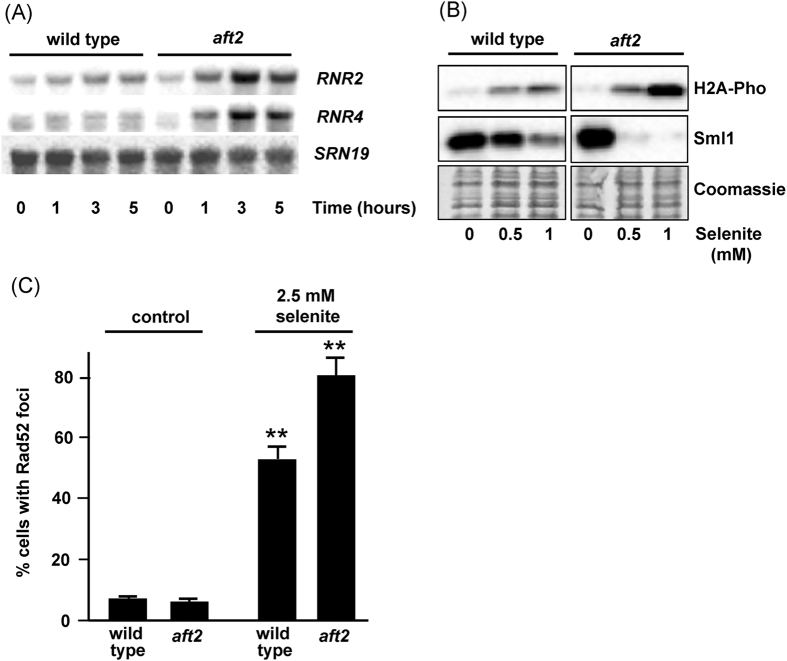
Cells lacking *AFT2* display increased DNA damage caused by sodium selenite. (**A**) Northern blot analysis of expression of the indicated genes in YPD medium cultures of wild type (W303-1A) and *aft2* (MML1086) cells after selenite addition (1 mM, final concentration) at time 0. *SNR19* is employed as loading and transfer control. (**B**) Western blot analysis of Sml1 and phosphorylated histone H2A levels in YPD medium cultures of wild type and *aft2* cells treated with the indicated concentrations of selenite for 4 hours. 15 μg of total protein were loaded per lane. Coomassie blue staining of a section of the blotted membrane is shown as loading control. (**C**) Wild type and *aft2* cells were transformed with plasmid pWJ1314 and grown in SC medium in the presence or absence of 2.5 mM selenite for 4 h. Cells with Rad52-YFP foci were counted and the results are expressed as the percentage over the total number of cells examined. Data are expressed as mean ± SD from three independent experiments. (Turkey-Kramer test, **p < 0.001 when compared with the control wild type value).

Clustering analysis (Supplementary Fig. S2) revealed that cluster 9 was enriched in genes involved in cellular response to stress (*p* < 6.8E-7), including diverse genes specifically induced by DNA damage, whose response was strongly potentiated in the *aft2* mutant (Fig. 2C). Other genes such as *RNR3*, *XRS2*, *MAG1*, *HUG1* and *DDR2*, known to encode proteins required for the cellular response to DNA damage and/or transcriptionally responding to this condition, were also remarkably induced in the Aft2-deficient strain. mRNA changes for *RNR2* and *RNR4* were monitored by Northern blot analysis (Fig. 3A), confirming that the expression of these genes became upregulated by exposure to selenite in *aft2* but not in wild type cells, with a ca. 4-fold peak over basal levels in the 3h-treatment samples of mutant cells. The *RNR* genes encode the subunits of ribonucleotide reductase, and they are induced by genotoxic agents through the DNA damage checkpoint pathway^33^. Therefore, our results suggested that the lack of Aft2 function increased selenite-provoked DNA damage. To confirm this hypothesis we determined histone H2A phosphorylation at Ser129, as marker of DNA damage^34^. No constitutive phosphorylation was observed in untreated cultures, but treatment with sublethal concentrations of selenite resulted in higher phosphorylation levels in the *aft2* mutant than in wild type cells (8-fold difference at 1 mM selenite) (Fig. 3B). Rad52 is a repair protein that binds DNA ends resulting from double or single strand breaks, and in response to DNA damage it localizes into discrete subnuclear foci^35^. We measured the presence of Rad52 foci in selenite-treated wild type and *aft2* strains. Treatment with this agent caused a significant increase in the frequency of Rad52-YFP foci, and this increase was more dramatic in the *aft2* mutant (Supplementary Fig. S3 and Fig. 3C), confirming that the genotoxicity of selenite is more intense in the absence of Aft2.

Dun1 kinase-mediated phosphorylation of the ribonucleotide reductase inhibitor Sml1 and its consequent degradation is a reliable marker of checkpoint activation as a result of DNA damage^33^,^36^,^37^. Sml1 levels were determined before and upon selenite treatment. Even at a selenite concentration as low as 0.5 mM a very pronounced reduction of Sml1 amount (at least 20-fold) was observed in *aft2* cells but not in the wild type, in which the reduction was only two-fold (Fig. 3B), denoting selenite-induced differential activation of the DNA damage checkpoint in the mutant cells. In contrast, no differential activation of the checkpoint seems to occur in untreated cultures. The above results clearly demonstrate that the *aft2* mutation is not genotoxic by itself but potently exacerbates the genotoxic effects of selenite.

### The *aft2* mutant overaccumulates selenium when exposed to selenite

**Figure 4:**
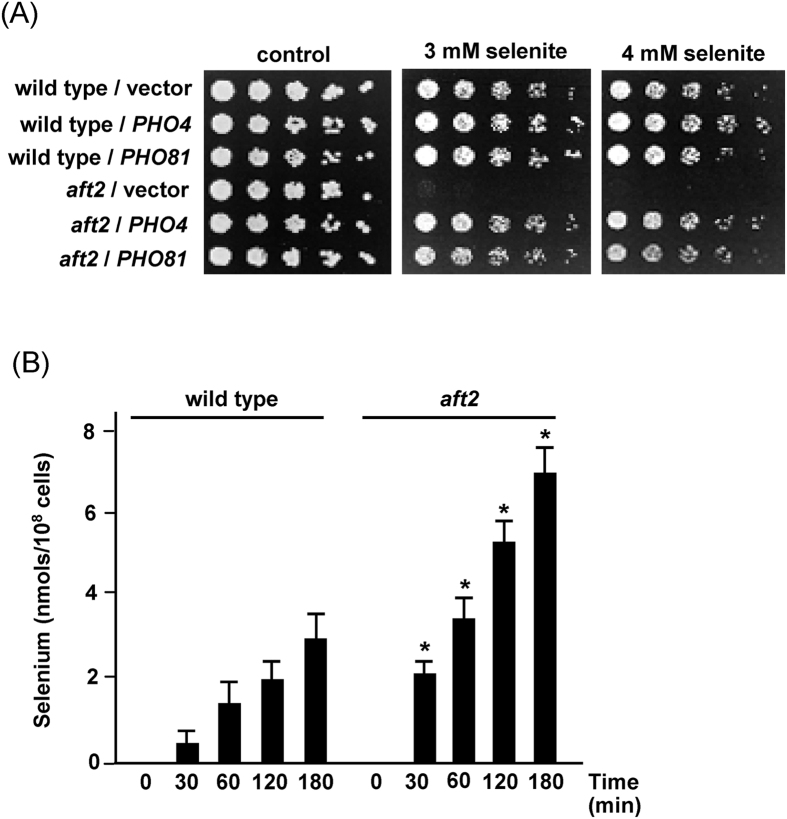
Cells lacking *AFT2* hyperaccumulate selenium compared to wild type cells. (**A**) Exponential cultures in SC medium of wild type (W303-1A) or *aft2* (MML1086) cells transformed with the multicopy plasmid YEplac181 (vector) or its derivatives pMM1102 (*PHO4*) or pMM1103 (*PHO81*) were serially diluted and spotted on SC plates with sodium selenite. Growth was recorded after 2 days at 30 °C. (**B**) Wild type and *aft2* cells growing exponentially in YPD medium were treated with 1 mM sodium selenite and samples taken at the indicated periods for determination of intracellular selenium accumulation by ICP-MS. Data are expressed as mean ± SEM from six independent determinations. (Turkey-Kramer test, *p < 0.05 when compared with the wild type value at the respective treatment time).

To advance in understanding how Aft2 could influence selenite toxicity in yeast cells, an *aft2* mutant was transformed with a yeast DNA overexpression library in the episomal vector YEp13 and clones were selected for growth in 2 mM selenite (see Materials and Methods). In addition to *AFT2*-containing plasmids, other four different plasmid inserts were found to rescue the hypersensitivity of the mutant (Supplementary Table S1). One of them (clone 4) included the *SSU1* and *GLR1* genes, which have already been demonstrated to increase tolerance to selenite when overexpressed in wild type cells^4^. In the case of the sulphite efflux pump *SSU1* gene^38^ the effect could be related to its additional role in selenite efflux^3^, while in the case of the glutathione reductase *GLR1* gene it could be related to counteracting the glutathione oxidation effects of selenite^6^,^8^. We have not further considered the function of *SSU1* and *GLR1* in relation to Aft2 it in the present study. Interestingly, the other plasmid clones were isolated several times each in the selection procedure and included two *PHO* genes related to phosphate transport and homeostasis: *PHO81* and *PHO4*. *PHO4* codes for the transcription factor required for expression of the *PHO* regulon under low environmental phosphate conditions, while *PHO81* encodes an inhibitor of the Pho80-Pho85 complex, consequently leading to nuclear translocation of Pho4 and transcription of the *PHO* regulon^12^. To confirm that *PHO4* and *PHO81* were responsible for selenite tolerance in *aft2* cells when overexpressed, we subcloned separately both genes plus their promoter regions in a multicopy vector and analysed the selenite sensitivity of the respective wild type and *aft2* transformants. Indeed, overexpression of *PHO4* or *PHO81* conferred selenite tolerance to the *aft2* mutant cells, while no significant effect was observed on wild type cells (Fig. 4A). To substantiate that under our working conditions overexpression of *PHO4* was inducing the *PHO* regulon, we determined the activity of the secreted repressible acid phosphatase Pho5, a typical readout of *PHO* activation. Indeed, we observed increases in Pho5 activity ranging from 12 to 30-fold in wild type and *aft2* cells, both in the absence or presence of 1 mM selenite, when compared to the same cells harbouring an empty vector (Supplementary Fig. S4). Therefore, these results indicate that activation of the *PHO* regulon have positive effects on selenite tolerance in Aft2-deficient cells.

High affinity phosphate transporters are members of the yeast *PHO* regulon, while the low affinity transporters are post-transcriptionally downregulated through this regulon (see Introduction). Given the role of both types of phosphate transporters in selenite uptake the above results suggest that selenite entry could be increased in Aft2-deficient cells with consequent intracellular accumulation of the compound. In fact, we demonstrated that Se overaccumulates inside *aft2* cells treated with a sublethal dosis of selenite (1 mM) compared to wild type cells (Fig. 4B), suggesting that alterations in selenite entry occur in the absence of Aft2. This overaccumulation could account for the hypersensitive phenotype of Aft2-deficient cells and explain the genotoxic effects previously observed.

### The low affinity phosphate transport system is altered in the absence of Aft2

In the growth conditions employed in the previous experiments (sufficient phosphate levels in the presence of glucose) selenite enters yeast cells mainly through the low affinity phosphate transport system^9^. The selenite hypersensitivity of *aft2* cells could therefore be an indication of upregulation of this transport system in the mutant. Spl2 is a post-transcriptional downregulator of the activity of the low affinity phosphate transporters Pho87 and Pho90^13^,^39^, although a more recent study^14^ has circumscribed this Spl2-mediated downregulation to Pho87. Since the *SPL2* gene is a member of the *PHO* regulon^11^, we hypothesised that overexpression of the *PHO* regulators *PHO4* or *PHO81* could result in increased levels of Spl2 and, therefore, in decreased activity of the Pho87-Pho90 transport system and consequent reduction of selenite entry, thus counteracting the effects of the absence of Aft2.

**Figure 5:**
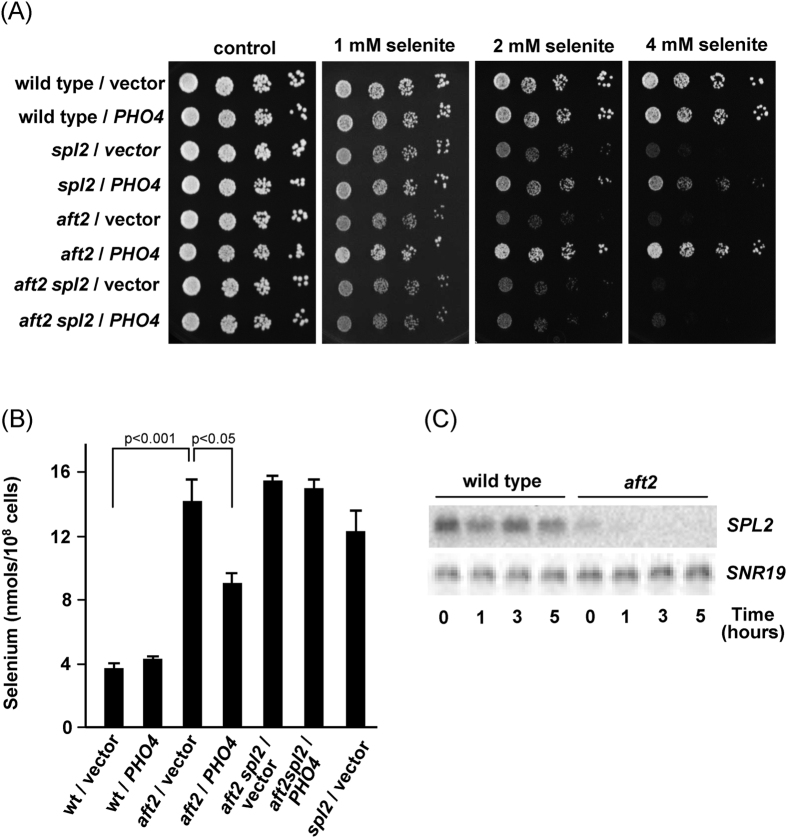
Spl2 is required for Pho2-mediated rescue of selenite hypersensitivity in *aft2* cells. (**A**) Exponential cultures in SC medium of wild type (W303-1A), *spl2* (ASC67), *aft2* (MML1086) and *aft2 spl2* (ASC69) cells transformed with the multicopy plasmid YEplac181 (vector) or its derivative pMM1102 (*PHO4*) were serially diluted and spotted on SC plates with sodium selenite. Growth was recorded after 2 days at 30 °C. (**B**) The indicated strains growing exponentially in SC medium were treated with 1 mM sodium selenite for 3 hours and samples were taken for determination of intracellular selenium by ICP-MS. Data are expressed as mean ± SEM from three independent determinations. (**C**) Northern blot analysis of expression of *SPL2* in YPD medium cultures of wild type and *aft2* cells after selenite addition (1 mM, final concentration) at time 0. *SNR19* is employed as loading and transfer control.

To test the former hypothesis, we determined whether (i) the absence of Spl2 was additive with that of Aft2 with respect to selenite hypersensitivity, and (ii) overexpression of *PHO4* was still able to suppress selenite hypersensitivity in the double *aft2 spl2* mutant. As already described^9^, deletion of *SPL2* resulted in increased susceptibility to selenite in an otherwise wild type background. However, this mutation did not further increase the hypersensitivity of Aft2-deficient cells (Fig. 5A), therefore supporting a linear relationship between Aft2 and Spl2. On the other hand, while overexpression of *PHO4* rescued the hypersensitivity to selenite in the single *aft2* mutant and at a lower extent also in the *spl2* mutant it did not in the double *aft2 spl2* mutant (Fig. 5A). These results correlated with the quantification of intracellular Se accumulation upon selenite addition to the medium. Thus, overexpression of *PHO4* diminished significantly the accumulation of selenium provoked by the absence of Aft2, and this reduction was Spl2-dependent (Fig. 5B).

Prompted by the above observations, we noticed that analysis of the transcriptome of *aft2* cells showed the downregulation of *SPL2* expression upon selenite treatment. We confirmed this result by Northern blot (Fig. 5C). Untreated *aft2* cultures already showed constitutive downregulation of *SPL2* mRNA levels compared to wild type cells (about 2.5-fold), and such levels became practically undetectable upon selenite treatment. This result indicates that Aft2 is necessary for proper expression of *SPL2* and suggests that, as a consequence, the lack of the transcription factor could increase the activity of the low affinity transport system resulting in enhanced transport of phosphate but also of selenite.

**Figure 6:**
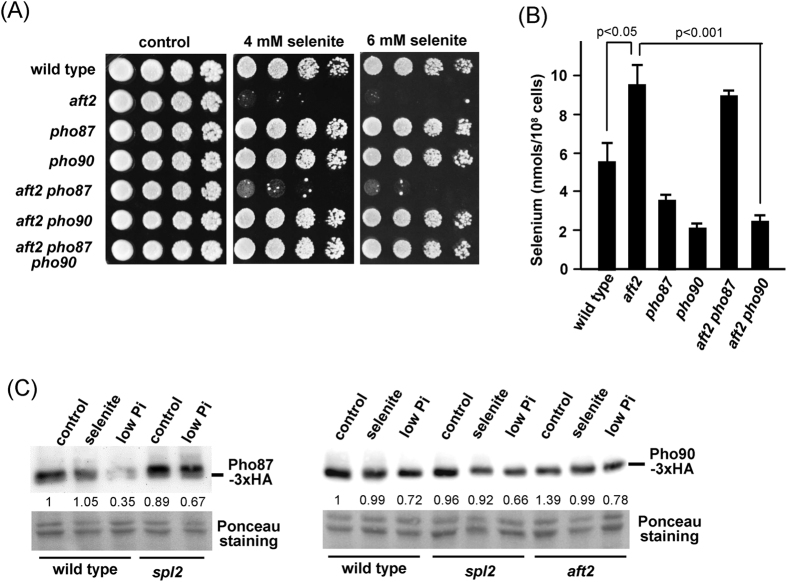
The low affinity phosphate transporter Pho90 is responsible for increased selenite toxicity in *aft2* cells. (**A**) Exponential cultures of the following strains in YPD medium were serially diluted and spotted on YPD plates containing sodium selenite: wild type (W303-1A), *aft2* (MML1086), *pho87* (MML2066), *pho90* (MML2068), *aft2 pho87* (MML2085), *aft2 pho90* (MML2088), and *aft2 pho87 pho90* (MML2095). Growth was recorded after 2 days of incubation at 30 °C. (**B**) The indicated strains growing exponentially in YPD medium were treated with 1 mM sodium selenite for 3 hours and samples were taken for determination of intracellular selenium by ICP-MS. Data are expressed as mean ± SEM from 3 to 12 independent determinations. (**C**) Western blot analysis of wild type (W303-1A), *spl2* (ASC67) and *aft2* (MML1086) cells transformed with plasmids pWS93-PHO87 (left panel) or pWS93-PHO90 (right panel), from cultures in standard SC medium without (control) or with 1 mM selenite (3 hours), or in low phosphate (0.1 mM) SC medium. Twenty-five μg of total protein were loaded per lane. Membranes were probed with anti-HA antibodies. Ponceau staining of sections of the blotted membranes is shown as loading control. Quantitative data of Pho87 or Pho90 levels (mean of three experiments) are indicated relative to the control (unit value), after being normalized by the respective Ponceau signals.

The next step was to determine whether Pho87 and Pho90 transporters are equally important for selenite uptake in the absence of Aft2. Deletion of *PHO90* in *aft2* cells rescued the hypersensitivity to selenite, while deletion of *PHO87* had only a marginal effect, and the triple mutant displayed a wild type phenotype (Fig. 6A). Mutation of *PHO84* did not alter sensitivity to selenite in the wild type or in the *aft2* strains (Supplementary Fig. S5, compare with Fig. 5A), indicating that this protein does not play a role in selenite transport under our study conditions. To confirm it, we analyzed the levels of the Pho84 protein in untreated and selenite-treated cells (Supplementary Fig. S6). These levels were lower in *aft2* cells than in wild type cells, and the *aft2* mutation was epistatic over the *spl2* one, corroborating that Aft2 acts upstream of Spl2. Importantly, the Pho84 levels were almost undetectable in selenite-treated *aft2* cells, discarding a significant participation in the transport of this compound.

The previous results support that both transporters Pho87 and Pho90 do not have an equivalent role in selenite transport when Aft2 is absent, and that in these conditions Pho90, but not Pho87, is competent for selenite entrance. The observations on selenite sensitivities correlated with measurements of intracellular Se accumulation in the respective strains. Thus, while *aft2* cells still accumulated Se in the absence of the Pho87 transporter, such accumulation was abrogated in the absence of Pho90, conditions in which intracellular Se levels were even lower than in wild type cells (Fig. 6B). To evaluate the possible impact of selenite on the amount of low-affinity transporters, we monitored the levels of HA-tagged versions of Pho87 and Pho90 by immunoblot. Treatment with selenite was without effect in wild type cells, whereas depletion of phosphate resulted in decreased amounts of Pho87, and this effect was abolished by deletion of *SPL2* (Fig. 6C). Given the genetic interaction between the *pho90* and *aft2* mutations concerning selenite sensitivity, in the case of the Pho90 protein the observations were extended to the *aft2* mutant. The amounts of Pho90 were essentially unaffected by phosphate starvation or addition of selenite irrespectively of the presence or absence of Spl2 or Aft2. Taking together, these experiments indicate that selenite toxicity on Aft2-deficient cells results from increased entry of the compound through the Pho90 transporter and that this occurs without significant changes in the amount of the transporter.

## Discussion

Although Aft1 and Aft2 have overlapping function in yeast cells, linked to iron assimilation, they also carry out regulatory functions that are specific of each of them. We show here a novel example of functional specificity for these transcription factors, since while cells lacking Aft1 or Aft2 are hypersensitive to selenite, this phenotype is rescued by iron addition only in the case of the *aft1* mutant, but not in *aft2* cells. That cells exposed to selenite require the Aft1 function to maintain constant intracellular levels of iron is confirmed by the significant iron depletion observed in selenite-treated *aft1* cells. This could be the consequence of direct interference of selenite with the mechanisms of iron uptake or derive indirectly from the intracellular oxidative stress generated by selenite^3^,^15^. Oxidant conditions downregulate the Fet4-mediated low affinity iron uptake system^40^, making therefore necessary the expression of the high affinity system, which requires the presence of Aft1. In any case, Aft2 can not substitute Aft1 in these functions, and the hypersensitivity of Aft2-deficient cells to selenite is independent of iron homeostasis. To our surprise deleting the *AFT1* gene in Aft2-less cells makes the selenite hypersensitivity of these cells to become in part iron-dependent (Fig. 1A). At this point we have no definite explanation for this result, although it suggests that Aft2 only acts as an additional co-control if Aft1 is present.

To advance in characterizing the relationship between Aft2 and selenite tolerance, we studied the transcriptome of selenite-treated *aft2* cells compared to wild type cells. About 60% of the genes upregulated in our study upon 60 min of treatment of wild type cells were also upregulated (more intensely in general) in the study of Salin *et al*.^15^. They are mostly involved in oxidative stress responses and iron uptake and homeostasis. On the contrary, the latter study observed a downregulating response in a large subset of genes that we were not able to observe. These differences could be due to the different methodologies employed or, more probably, to the fact that although the same selenite dose (1 mM) was applied in both cases, the BY4742 strain utilized by Salin *et al*. is clearly more sensitive to selenite than the wild type strain employed in this work. In fact, in our genetic background, this selenite concentration does not result in measurable growth rate decrease. Importantly, the *aft2* mutation strongly potentiates many changes observed in the wild type strain, suggesting a stronger impact of this low dose of selenite in the physiology of the cell. This notion is supported by our observation that selenite causes exacerbated genotoxic effects in the *aft2* mutant (Fig. 3). Thus, DNA damage is a good reporter of selenite levels in the cell and the toxic effects of this agent.

In sufficient-phosphate and glucose-rich medium (our experimental conditions), selenite uptake occurs through the low-affinity phosphate uptake system^9^. The identification in our suppressor screen of *PHO81* and *PHO4*, two genes that positively influence high-affinity phosphate transport and, in parallel, downregulate low-affinity uptake, made us to focus attention on the possible influence of Aft2 on selenite transport through the Pho87/Pho90 low-affinity system. We indeed show (Fig. 4B) that Aft2-deficient cells overaccumulate selenium, thus suggesting that Aft2 might influence the function of that system. Remarkably, the analysis of tolerance to selenite in *aft2* mutants lacking *PHO87* or/and *PHO90* shows that the contribution of Pho87 must be only marginal, since deletion of *PHO87* improves only slightly selenite tolerance in the *aft2* mutant background, whereas, in contrast, deletion of *PHO90* practically normalizes tolerance to selenite (Fig. 6A). This notion is confirmed by the observation that deletion of *PHO90* greatly decreases intracellular selenium both in wild type and in *aft2* cells, whereas that of *PHO87* does not. Additionally, the very low levels of Pho84 in selenite-treated *aft2* mutant cells support that the high-affinity phosphate transport system does not participate in selenite uptake in these conditions. Also, the decrease in expression of diverse genes belonging to the *PHO regulon* observed in our RNA-seq analysis (Supplementary Fig. S2), together with the lower expression of Pho84 in *aft2* cells compared to wild type cells even in untreated cultures (Supplementary Fig. S6), and the lower levels of Pho5 activity in Aft2-deficient cells (Supplementary Fig. S4) are observations that point towards a general mechanism for Aft2 in regulating the *PHO* regulon. Altogether, our results suggest that in our experimental conditions selenite enters mainly through the Pho90 transporter, in agreement with the reported observation that, when sufficient amounts of phosphate are available, Pho90 becomes the most relevant phosphate transporter^14^. The observations that *JEN1* expression is strongly repressed by glucose^41^ and that resistance to selenite is not altered by deletion of *JEN1* in cells grown with glucose as a carbon source^10^, allow discarding any contribution of Jen1 in selenite transport under our study conditions.

There is some controversy about the mechanism controlling stability of Pho87 and Pho90 and its vacuolar degradation fate. A role for Pho4-mediated control of Spl2 levels, which in turn would negatively control low-affinity phosphate uptake, seems clear. However, discrepancies exist about the underlying mechanism, and it has been proposed that it affects Pho87 and Pho90^13^,^39^ or only Pho87^14^. It is worth noting that we have been able to confirm by immunoblot a Spl2-dependent decrease in the amount of protein for Pho87 (but not for Pho90) in response to phosphate depletion.

**Figure 7:**
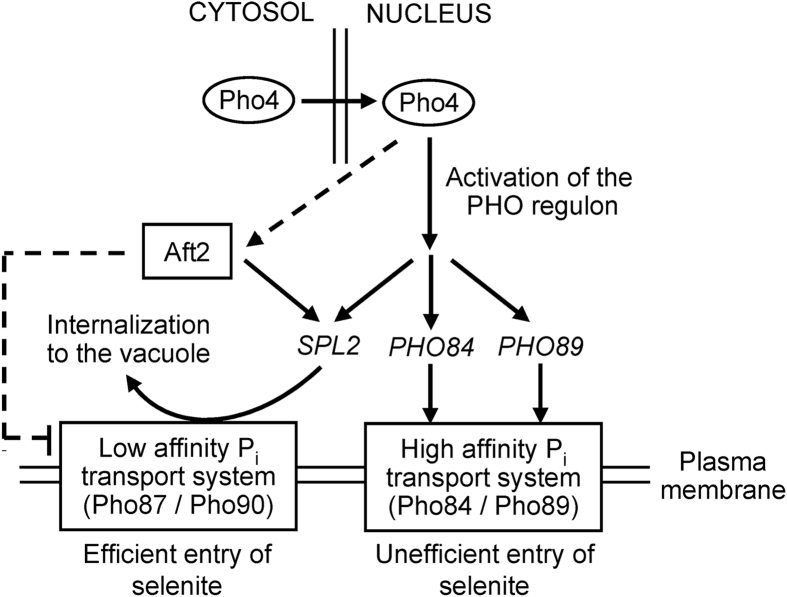
Scheme of the proposed relationship between Aft2 and the phosphate and selenite transport systems. See the main text for details. Dashed lines represent hypothetical relationships raised from this work.

We show that overexpression of *PHO4* substantially improves tolerance and reduces selenium content of the *aft2* strain. Given the major role of Pho90 in selenium toxicity, this suggests that, indeed, Spl2 is controlling Pho90 function. We have confirmed the previous observation by Lazard *et al*.^9^ that a *spl2* mutant is sensitive to selenite, and we also show here that this effect is alleviated by overexpression of *PHO4*, similarly to what is observed for the *aft2* strain (Fig. 5A). This is an interesting observation, because it suggests that there must be a Spl2-independend, Pho4-responsive process with relevance on selenite toxicity. Also remarkable is the observation that the absence of Aft2 results in lower-than-normal expression of *SPL2*, and this effect is enhanced by exposure to selenite, leading to undetectable levels of *SPL2* mRNA (Fig. 5C). This observation reinforces that Aft2 positively regulates the expression of members of the *PHO* regulon. We present in Fig. 7 a working model that could explain our results on the light of the existing knowledge. The downregulation of *SPL2* expression in *aft2* mutant cells and the increase of Se levels in the *spl2* mutant argue in favour of the involvement of Spl2 levels in the selenite hypersensitivity of *aft2* cells. The fact that the overexpression of *PHO4* reduces Se accumulation and toxicity in a Spl2-dependent manner also supports such involvement of Spl2 in the sensitivity of the *aft2* cells. However, it is worth noting that, since overexpression of *PHO4* still improves tolerance in the *spl2* mutant but no longer does in the double *aft2 spl2* strain (Fig. 5A) it can be conceived the existence of a Pho4-dependent but Spl2-independent (and likely, Aft2-mediated) component in selenite tolerance. Such notion would be consistent with the proposal by Ghillebert *et al*.^14^ in that other factors besides Spl2 would also mediate endocytosis of both Pho90 and Pho87. It must be noted that our results do not allow explaining the increased sensitivity of the *aft2* mutant on the basis of increased total amounts of Pho90 (Fig. 6C). Therefore, it is conceivable to assume that lack of Aft2 might result in relocalization of Pho90 at the cell membrane, increased activity of the transporter and/or gain of specificity for selenite against phosphate.

## Methods

### Strains, plasmids and growth conditions

The strains employed in this study (W303 genetic background) are listed in Table 1. Plasmid pWJ1314 expresses the Rad52-YFP construction under the control of the own *RAD52* promoter^42^. Plasmid pMM1102 contains the *PHO4* ORF plus adjacent regions (from −788 from the initiating Met to +336 from the stop codon), cloned between the EcoRI-BamHI sites of multicopy vector YEplac181^43^. The pMM17-PHO84 plasmid carries the *PHO84* gene (expressed from its own promoter) fused with the 3x-HA epitope^44^. Plasmid pMM1103 contains the *PHO81* ORF plus adjacent regions (from −684 to +342) cloned between the KpnI-SalI sites of YEplac181. Plasmid-borne expression of 3x-HA N-terminally tagged versions of Pho87 and Pho90 were accomplished by amplification of the corresponding ORFs with the oligonucleotide pairs 5_PHO87_EcoRI/3_PHO87_BamHI, and 5_PHO90_EcoRI/3_PHO90_BamHI, respectively (Supplementary Table S2). The amplification fragments were digested with EcoRI and BamHI and cloned into the same sites of plasmid pWS93 (multicopy, *URA3* marker) to allow expression from the *ADH1* promoter^45^, resulting in plasmids pWS93-PHO87 and pWS93-PHO90 respectively.

YPD (1% yeast extract, 2% peptone, 2% glucose) or SC medium^46^ were usually employed for *S. cerevisiae* cell growth. Media were solidified with 2% agar. Sodium selenite (Sigma) was added at the concentrations indicated in each case to mid-exponential cultures (about 1−2 × 10^7^ cells per ml). Cells were grown at 30 °C, with shaking in the case of liquid cultures. Sensitivity to selenite was determined in plate growth assays by spotting serial 1:5 dilutions of the respective strain cultures onto plates with solid medium containing this agent, and recording growth after 2 or 3 days of incubation at 30 °C. Phosphate starvation conditions were generated by growing cells in SD broth with 2% glucose w/o phosphate (Formedium) to which KH_2_PO_4_ was added at 0.2 mM (final concentration).

### Genetic methods

Standard protocols were used for DNA manipulations and transformation of yeast cells. Single null mutants in which the entire open reading frames were removed were generated using the short-flanking homology approach after PCR amplification of the *kanMX4*^47^, *natMX4*^48^ or *his3MX6*^49^ and selection for G418 or nourseothricin resistance, or in the absence of histidine, respectively. Multiple mutants were obtained by crossing the parental mutant strains, followed by diploid sporulation, tetrad analysis or random spore analysis, and selection of the mutant combinations^44^ with the exception of strain ASC69 in which the *SPL2* gene was disrupted by short-flanking homologous recombination using a *HIS3MX6* cassette, amplified from plasmid pFA6a-HISMX6^49^ with oligonucleotides SPL2-5-NAT and SPL2-3-NAT. Disruptions were confirmed by PCR analysis. Oligonucleotides employed in this study are indicated in Supplementary Table S2.

### Isolation of suppressors of the selenite hypersensitivity of *aft2* cells

Exponentially growing MML1086 (*aft2*) cells in YPD medium were transformed with a yeast genomic DNA library in the multicopy plasmid YEp13^50^, using the lithium acetate method^51^. Transformants were selected in SC medium plates containing 3 mM sodium selenite plus 0.1 mM ferrous sulfate/0.25 mM ferrozine. Addition of these latter components increased transformation efficiency about 3-fold. Parallel plating was done on medium without selenite to quantify transformation efficiency. Several independent experiments were done to amount a total of about 40,000 transformants on non-selenite medium. Plasmids were recovered from *aft2* clones growing in the above selective conditions, amplified in *Escherichia coli* and retransformed on MML1086 cells to confirm the ability to suppress the selenite hypersensitivity of *aft2* cells. Finally, positive plasmids were grouped according to their *EcoR*I-*Hind*III restriction pattern. In addition to *AFT2*-containig plasmids, four different groups were established in this way, and one representative clone was selected from each group for sequencing the ends of the yeast DNA inserts and consequently determining the yeast genes present in each clone.

### Transcriptomic analysis by RNA-Seq

Wild type W303-1A and its MML1086 derivative (*aft2*) were grown in YPD medium until 1 × 10^7^ cells/ml. Sodium selenite was then added to reach a final concentration of 1 mM and growth was continued. Cultures were rediluted in selenite-containing medium when required, to avoid reaching concentrations higher than 5 × 10^7^ cells/ml. Aliquots of the culture (6 × 10^8^ cells) were taken after 1, 3 and 5 h of selenite treatment, and cells collected and processed for total RNA isolation as in Bellí *et al*.^52^. Poly A (+) RNA was selected with the NEBNext^®^ Poly(A) mRNA Magnetic Isolation Module (New England Biolabs, Ref. E7490). Preparation of libraries was carried out with NEBNext^®^ Ultra™ Directional RNA Library Prep Kit (Ref. E7420) and the Illumina oligo set 1 (Ref. E7335). Sequencing was performed in an Illumina MiSeq machine and the MiSeq Reagent Kit v3 (single end, 150 nt/read). Two biological replicates were done.

Mapping of fastq files to generate SAM files was carried out with Bowtie2 software^53^ in end-to-end mode. The number of mapped reads ranged from 4.96 to 6.01 million per sample. The SAM files were analyzed with the SeqMonk software (http://www.bioinformatics.bbsrc.ac.uk/projects/seqmonk). Mapped reads were counted using CDS probes (extended 50 nt upstream and downstream) and corrected for the largest dataset. Data can be retrieved from the Gene Expression Omnibus (GEO) repository under study GSE70835.

### Northern blot analyses

RNA isolation and electrophoresis, probe labelling with digoxigenin, hybridization, and signal detection and quantification were done as described previously^52^. Gene probes were generated by PCR from genomic DNA, using oligonucleotides designed to amplify internal open reading frame sequences. *SNR19* mRNA was employed as loading control.

### β-galactosidase reporter assays

Determination of the transcriptional activity of *GRE2*, *TRX2* and *GRX1* was carried out by generating transcriptional fusions of the respective promoters with the *lacZ* gene. To this end, upstream regions of *GRE2* (positions −1178/+26 nt respective the initial Met codon), *TRX2* (−981/+11) and *GRX1* (−851/+33) were amplified from genomic DNA with the appropriate oligonucleotides described in Supplementary Table S2. The amplified fragments were cloned into the KpnI-HindIII sites (*GRE2*), or EcoRI-HindIII (*TRX2* and *GRX1)* of plasmid YEp357^54^. β-galactosidase activity was determined in permeabilized cells as in Ruiz *et al*.^55^.

### Western blot analyses

Western blot analyses and signal quantification were done as described in Bellí *et al*.^56^, with the following exceptions: for monitoring the HA-tagged versions of Pho87 and Pho90 extracts were prepared as described in Serra-Cardona *et al*.^57^. For evaluation of HA-tagged Pho84 levels, the procedure described by Canadell *et al*.^44^ was followed. Rabbit anti-histone H2A (phosphoS129) (from Abcam, dilution 1:1,000), rabbit anti-Sml1 (from Agrisera, dilution 1:1,000) and, for HA-tagged proteins, the mouse monoclonal anti-HA antibody 12CA4 (Roche, dilution 1:1,000) or the rabbit polyclonal anti-HA antibody (Abcam, #ab9110, dilution 1:4,000) were employed as primary antibodies.

### Quantification of Rad52-YFP foci

Cells growing in SC, transformed with plasmid pWJ1314 expressing Rad52-YFP, were observed with an Olimpus BX51 fluorescence microscope equipped with an Olimpus DP30BW digital camera, using excitation and emission wavelengths of 480 and 527 nm respectively. Foci were inspected by examining all of the focal planes intersecting each cell. Three independent experiments were performed for each strain and condition, and at least 500 cells were counted per sample.

### Determination of selenium accumulation

All plastic ware used was soaked overnight with 10% nitric acid and subsequently thoroughly rinsed with MilliQ water at least five times. Cultures were grown in YPD until 2.5 × 10^7^ cells per ml and sodium selenite added up to 1 mM. Samples (10 ml or the equivalent volume to yield 2.5 × 10^8^ cells) were taken at 0, 30, 60, 120 and 180 min, and rapidly vacuum filtered on Omnipore membrane filters (0.2 μm pore size, Millipore). Filters were washed once with 12 ml and twice with 5 ml of 1 M EDTA Na_2_ (pH 8.0) solution, and then three times with 5 ml ice-cold MilliQ water. Filters were then placed on screw-cap microcentrifuge tubes, 500 μl of 30% nitric acid (Ensure^®^ ISO, #1.000456, Merck-Millipore) was added and samples were digested overnight in a 65 °C water bath. Then, 500 μl of MilliQ water was added, samples were briefly vortexed, centrifuged (10 min, 12,000 × g) and 950 μl of the supernatants transferred to fresh tubes for Se determination. The accumulation of Se was measured in samples diluted in 1% (v/v) nitric acid by Inductively Coupled Plasma Mass Spectrometry (ICP-MS), using an Agilent 7500 ce apparatus. The values obtained were corrected by subtracting the nearly negligible background determined at time = 0.

### Other methods

Intracellular iron was determined as in Tamarit *et al*.^58^. Determination of extracellular Pho5 activity in liquid cultures was done as in Huang and O’Shea^59^.

## Additional Information

**How to cite this article**: Pérez-Sampietro, M. *et al*. The yeast Aft2 transcription factor determines selenite toxicity by controlling the low affinity phosphate transport system. *Sci. Rep.* **6**, 32836; doi: 10.1038/srep32836 (2016).

## Supplementary Material

Supplementary Information

## Figures and Tables

**Table 1:** Strains employed in this study.

Strain	Genotype	Source and comments
W303-1A	*MAT*a *ura3-1ade2-1 leu2-3,112 trp1-1 his3-11,15 can1-1*	Wild type
MML348	W303-1A *aft1-Δ5::URA3*	Ref. 29
MML1086	W303-1A *aft2::kanMX4*	Ref. 29
MML1088	W303-1A *aft1-Δ5::URA3 aft2::kanMX4*	Ref. 29
MML1304	W303-1A *pho84::natMX4*	This work
MML1748	W303-1A *gex1::natMX4*	This work
MML1750	W303-1A *gex2::kanMX4*	This work
MML1752	W303-1A *gex1::natMX4 gex2::kanMX4*	This work
MML2054	W303-1A *aft2::kanMX4 pho84::natMX4*	This work
MML2066	W303-1A *pho87::natMX4*	This work
MML2068	W303-1A *pho90::natMX4*	This work
MML2085	W303-1A *aft2::kanMX4 pho87::natMX4*	This work
MML2088	W303-1A *aft2::kanMX4 pho90::natMX4*	This work
MML2095	W303-1A *aft2::kanMX4 pho87::natMX4 pho90::natMX4*	This work
ASC67	W303-1A *spl2::his3MX6*	This work
ASC69	W303-1A *aft2::kanMX4 spl2::his3MX6*	This work
