# Supplementary material for: The yeast Aft2 transcription factor determines selenite toxicity by controlling the low affinity phosphate transport system
Source: Sci Rep. 2016 Sep 13;6:32836. doi: 10.1038/srep32836 (PMC5020356; doi:10.1038/srep32836)
Supplement: Supplementary Information [file srep32836-s1.pdf]

Supplementary Information

The yeast Aft2 transcription factor determines selenite toxicity by controlling the low affinity phosphate transport system

María Pérez-Sampietro, Albert Serra-Cardona, David Canadell, Celia Casas, Joaquín Ariño and Enrique Herrero

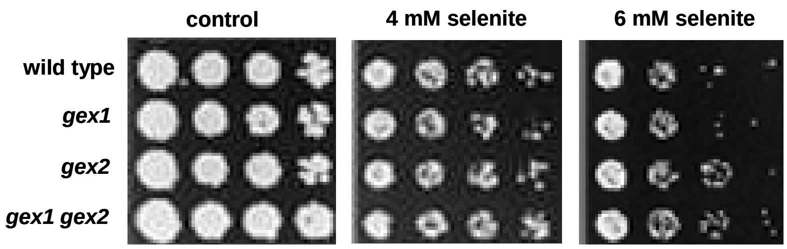

Supplementary Fig. S1. Exponential cultures of the following strains in YPD medium were serially diluted and spotted on YPD plates containing sodium selenite: wild type (W303-1A), *gex1* (MML1748), *gex2* (MML1750) and *gex1 gex2* (MML1752). Growth was recorded after 2 days of incubation at 30ºC.

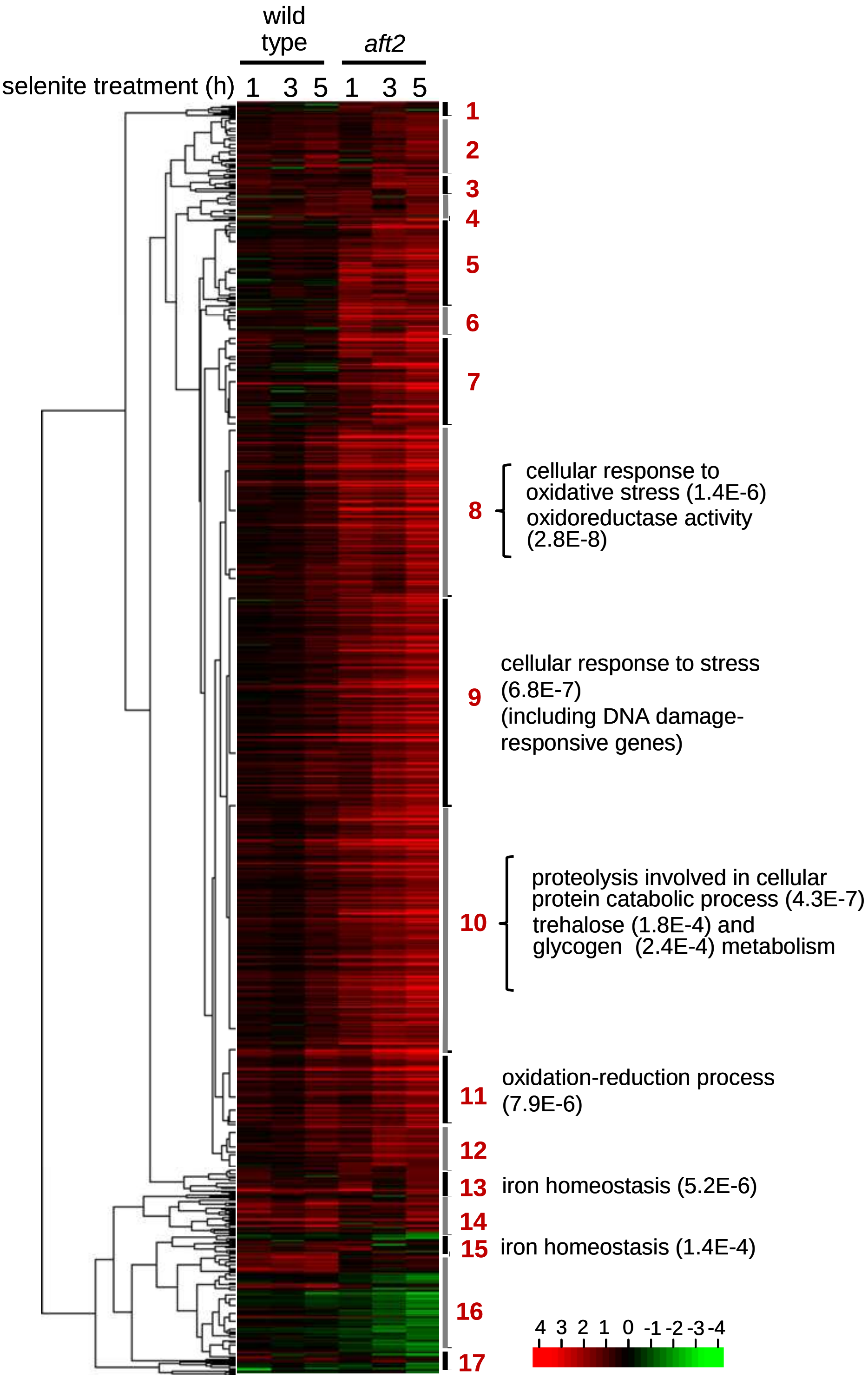

Supplementary Fig. S2. Clustering analysis of differentially expressed genes in wild type (W303-1A) and *aft2* (MML1086) cells upon 1 mM selenite treatment for the indicated times. A total number of 968 genes with at least 2-fold change for at least 1 time-point were grouped with the Cluster 3.0 software (de Hoon, M.J. *et al.* *Bioinformatics* **20,** 1453-1454, 2004) (Spearman Rank Correlation, average linkage) and results represented with the Java Treeview software, version 1.1.6r4 (Saldanha, A.J. *Bioinformatics* **20,** 3246-3248, 2004). Functional categories significantly enriched in the corresponding clusters are indicated. A reference bar (log2 scale) is included at the right bottom.

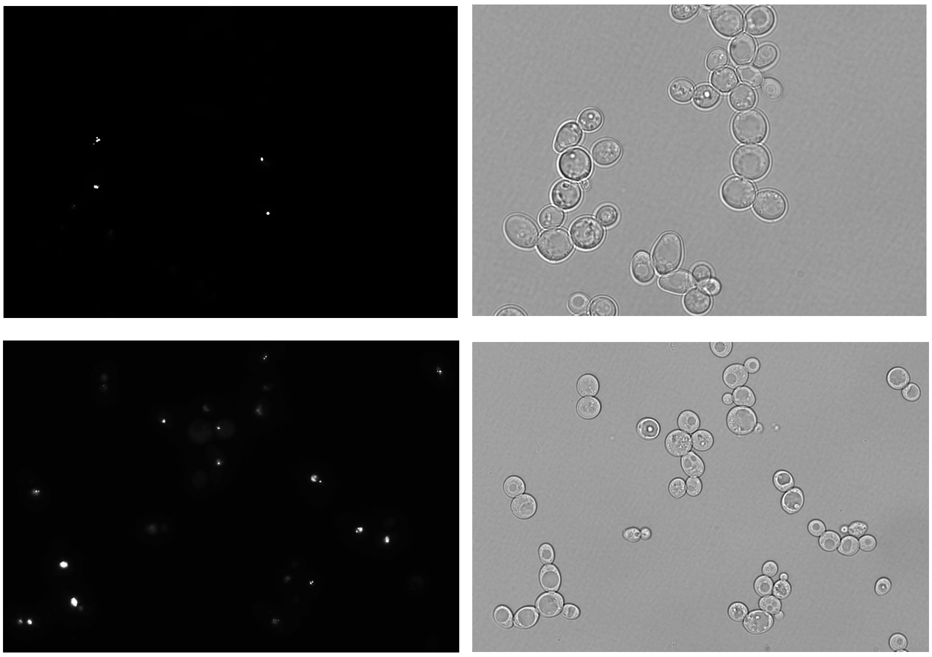

Supplementary Fig. S3. Left panel: Rad52-YFP foci in plasmid pWJ1314-transformed wild type (W303-1A) and *aft2* (MML1086) cells treated for 4 hours with 2.5 mM selenite in SC medium. Right panel: phase constrast (PC) microscopy image of the same field.

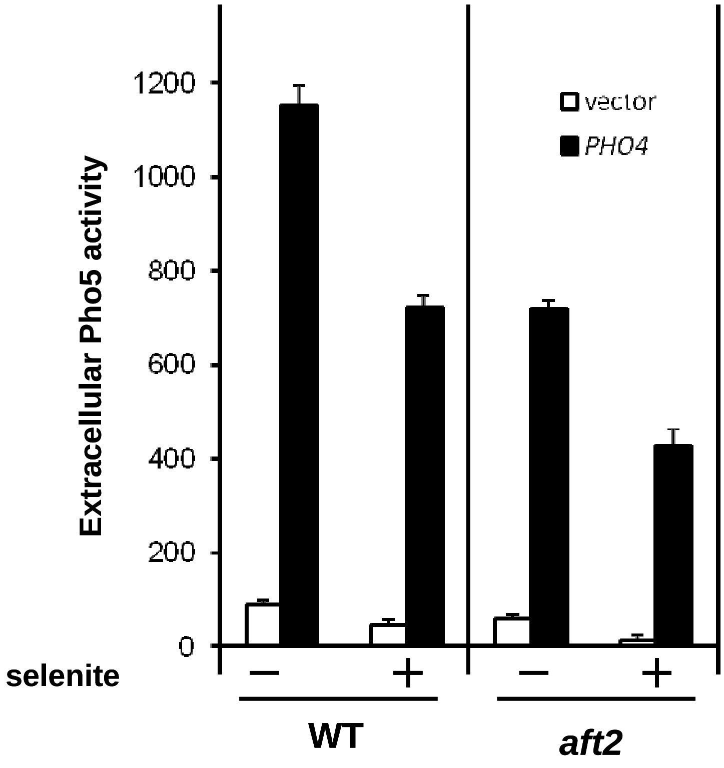

Supplementary Fig. S4. Pho5 activity upon transformation of wild type (WT, W303-1A) and *aft2* (MML1086) cells with multicopy plasmid YEplac181 (vector) or its derivative pMM1102 (*PHO4*). Cultures were grown overnight in SD media lacking leucine and inoculated into YPD media at OD_{600} of 0.2. When cultures reached the exponential phase selenite was added (final concentration 1 mM) and growth resumed for 3 h. After this period Pho5 activity in the medium was measured. Data are mean ± SD from 3 experiments.

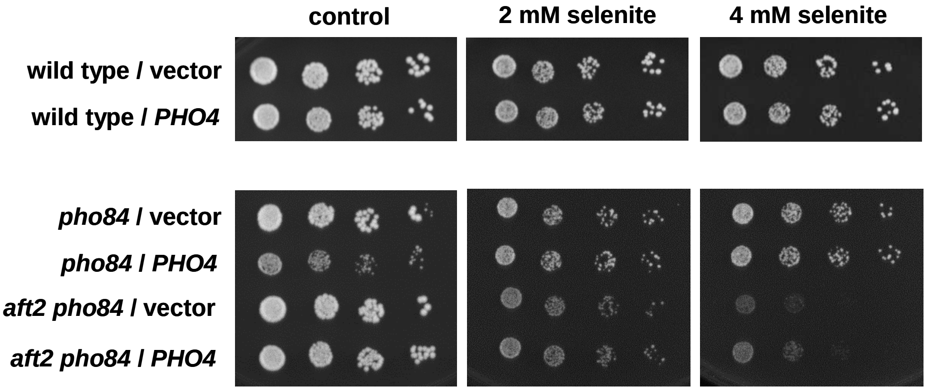

Supplementary Fig. S5. The *pho84* mutation does not alter selenite sensitivity. Exponential cultures in SC medium of wild type (W303-1A), *pho84* (MML1304), and *aft2 pho84* (MML2054) cells transformed with the multicopy plasmid YEplac181 (vector) or its derivative pMM1102 (*PHO4*) were serially diluted and spotted on SC plates with sodium selenite. Growth was recorded after 2 days at 30ºC.

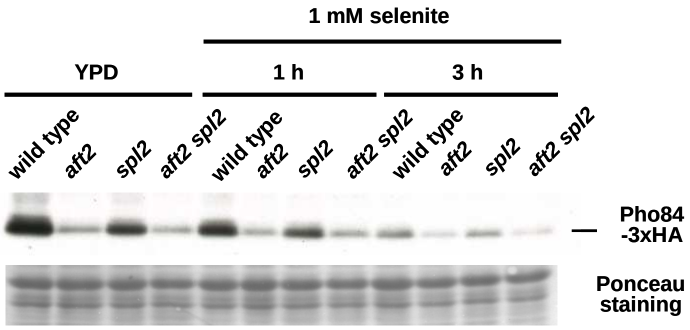

Supplementary Fig. S6. Pho84 protein levels in selenite-treated cells. Western blot analysis of extracts from wild type (W303-1A), *aft2* (MML1086), *spl2* (ASC67) and *aft2 spl2* (ASC69) cells transformed with pMM17-PHO84 plasmid carrying the *PHO84* gene fused with the 3x-HA epitope. Cells were grown in YPD medium to exponential phase, subjected to selenite (1 mM) stress and collected after the indicated times. Forty µg of total protein were loaded per lane. Membranes were probed with anti-HA antibodies. Ponceau staining of sections of the blotted membranes is shown as loading control.

Supplementary Table S1. Properties of the YEp13 overexpression library clones able to suppress the selenite hypersensitivity of *aft2* cells

Clon	Chromosome	Coordinates of the insert ends	Insert genes
1	VII	954258 / 959673	[*NAS6*] / *PHO81*
2	VI	221646 / 227939	*RUF23* / *RRT5* / *RPL29* / *YFR032C-B* / *QCR6* / *PHO4* / *YFR035C* / *CDC26* / [*RSC8*]
3	VII	953474 / 959441	*NAS6* / *PHO81*
4	XVI	371742 / 377805	[*NOG1*] / *SSU1* / *GLR1* / [*RPS6A*]

Genes truncated in the insert are into brackets

Supplementary Table S2. Oligonucleotides employed in this study

Name	Sequence	Use
GRE2_prom_5'	gcggtaccgtcggaaattccgtggc	Amplification of *GRE2* promoter for construction of pGRE2-LacZ
GRE2_prom_3'	cgaagcttagcacctgaaacgaaaac	Amplification of *GRE2* promoter for construction of pGRE2-LacZ
TRX2_prom_5'	gcgaattcggtgggtacggttc	Amplification of *TRX2* promoter for construction of pTRX2-LacZ
TRX2_prom_3'	gcaagctttaattgagtgaccattattg	Amplification of *TRX2* promoter for construction of pTRX2-LacZ
GRX1_prom_5'	gcgaattcttaataatcctaggctc	Amplification of *GRX1* promoter for construction of pTRX2-LacZ
GRX1_prom_3'	gcaagcttgacgtgcttgatagtttcttg	Amplification of *GRX1* promoter for construction of pTRX2-LacZ
MMO1781	aaaggaggtacccttgcttgtgctatctcaag	Upstream primer for subcloning of *PHO81* plus adjacent regions in YEplac181
MMO1782	aaaggagtcgacatggtgcgtccgtaagaa	Downstream primer for subcloning of *PHO81* plus adjacent regions in YEplac181
MMO1783	aaaggagaattcatcggtagcgcgtatgac	Upstream primer for subcloning of *PHO4* plus adjacent regions in YEplac181
MMO1784	aaaggaggatcccatgctgctgctcgttatcgt	Downstream primer for subcloning of *PHO4* plus adjacent regions in YEplac181
MMO1664	agttttatggactggaaagagaaaaaaaaaaaaaag gaataatcaagtaacgtacgctgcaggtcgac	Upstream primer for disruption of *GEX1* with the *MX* cassettes
MMO1665	tcaccagcttggggatcaacatcctaatttcttgttacttcg attatgtcatcgatgaattcgagctcg	Downstream primer for disruption of *GEX1* with the *MX* cassettes
MMO1668	agttttatggactggaacgagaaaaaaaaaaaaaag aataatcaagtaaatcgtacgctgcaggtcgac	Upstream primer for disruption of *GEX2* with the *MX* cassettes
MMO1669	tcaccagcttggggatcaacatcctgatttcttgttacttcg attatgtcatcgatgaattcgagctcg	Downstream primer for disruption of *GEX2* with the *MX* cassettes
MMO1997	taaaggtgcagcaaaactacggtaaaggaggctattat tgtgcctcgaacgtacgctgcaggtcgac	Upstream primer for disruption of *PHO87* with the *MX* cassettes
MMO1998	aaaggtacaaagaatgcagtaataagaaatgtaatta catgagggcagtatcgatgaattcgagctcg	Downstream primer for disruption of *PHO87* with the *MX* cassettes
MMO2001	taaagttgtttttaggataaacgagtaagtggtagctggt acaggatccgtacgctgcaggtcgac	Upstream primer for disruption of *PHO90* with the *MX* cassettes

MMO2002	tacatatagattatatatacgctgttacatgctatcgtaga cgtatctcatcgatgaattcgagctcg	Downstream primer for disruption of *PHO90* with the *MX* cassettes
SPL2-5-NAT	cactgcagccacgtgcctagatctattactatgacttccc cgtacgctgcaggtcgac	Upstream primer for disruption of *SPL2* with the *MX* cassettes
SPL2-3-NAT	ttcgaatatgtcttcgtcaatgacacagtcgttctcccaaa tcgatgaattcgagctcg	Downstream primer for disruption of *SPL2* with the *MX* cassettes
5_PHO87_EcoRI	cggaattcatgagattctcacactttctcaaa	Amplification of *PHO87* for construction of pWS93-PHO87
3_PHO87_BamHI	cgggatccttaagtgctaccttttaagacgga	Amplification of *PHO87* for construction of pWS93-PHO87
5_PHO90_EcoRI	cggaattcatgagattttcacacttcttgaag	Amplification of *PHO90* for construction of pWS93-PHO90
3_PHO90_BamHI	cgggatccttacgcagaggttgcgttac	Amplification of *PHO90* for construction of pWS93-PHO90
